# Enlarged Prostate

**Published:** 1904-07-23

**Authors:** 


### ENLARGED PROSTATE.

The large measure of success which has recently attended the radical treatment of protastic enlargements, and the obvious fact that the chances of recovery are enhanced by operating before secondary complications have ensued, are leading to a widespread tendency to prefer early radical measures to the old and more general plan of temporising with the catheter until some symptom of danger appears. Meyer[1] is of opinion that as soon as it is necessary to begin "catheter life" it is time for operative intervention to be considered. For the treatment of enlarged prostate the catheter is only justified in the well-to-do and intelligent classes, where neither the physician nor the patient is willing to face operation, and in people suffering from advanced diabetes (5 per cent. of sugar), chronic myocarditis and contracted kidney. In the case of men who have to work for their living, it is only after they decline operation of every kind that the catheter should be offered them. Prostatectomy is the most satisfactory operation for relieving the obstruction to the outflow of urine, and its mortality, Meyer says, is but 5 per cent. If the patient's condition or wishes will not allow prostatectomy, then the galvano-caustic prostatotomy may be resorted to. Prostatectomy is contra-indicated where there is pronounced anæmia, very soft gland, or marked contracture of the hips. The age limit should be extended indefinitely, and, if the patient will not stand a general anæsthetic, spinal anæsthesia may be resorted to. Meyer prefers the perineal to the supra-pubic form of operation, making Dittel's[2] pre-anal incision. He claims that thus the surgeon works less in the dark, and that soft glands with hard nuclei can be easily extracted, whereas through the supra-pubic wound this is difficult; unless there are signs of kidney trouble the perineal operation should be preceded by a cystoscopic examination, for in 33 per cent. of the cases of enlarged prostate the tumour is not discernable by the finger in the rectum, and the cystoscope reveals the configuration of the gland, the condition of the bladder, and the presence or absence of calculi. With regard to the effect of operation on the sexual powers he says that in 86 per cent. of his patients, after prostatomy, it is unaffected. After prostatectomy it is more often affected, but definite observations have not been made. Lilienthal,[3] on the other hand, claims that rarely, if ever, does impotency follow supra-pubic prostatectomy.

[1] Medical Record, June 25th, 1904. [2] Wiener medizinische Wochenschrift, 1874, No. 16. [3] Medical News, June 25th, 1904.

